# Effect of transcranial direct current stimulation on the functionality of 40 Hz auditory steady state response brain network: graph theory approach

**DOI:** 10.3389/fpsyt.2023.1156617

**Published:** 2023-06-09

**Authors:** Tetsu Hirosawa, Daiki Soma, Yoshiaki Miyagishi, Naoki Furutani, Yuko Yoshimura, Masafumi Kameya, Yohei Yamaguchi, Ken Yaoi, Masuhiko Sano, Koji Kitamura, Tetsuya Takahashi, Mitsuru Kikuchi

**Affiliations:** ^1^Research Center for Child Mental Development, Kanazawa University, Kanazawa, Japan; ^2^Department of Psychiatry and Neurobiology, Graduate School of Medical Science, Kanazawa University, Kanazawa, Japan; ^3^Faculty of Education, Institute of Human and Social Sciences, Kanazawa University, Kanazawa, Japan; ^4^Department of Neuropsychiatry, Faculty of Medical Sciences, University of Fukui, Fukui, Japan

**Keywords:** auditory steady state response, transcranial direct current stimulation, magnetoencephalography, graph theory, functional brain network

## Abstract

**Introduction:**

Measuring whole-brain networks of the 40 Hz auditory steady state response (ASSR) is a promising approach to describe the after-effects of transcranial direct current stimulation (tDCS). The main objective of this study was to evaluate the effect of tDCS on the brain network of 40 Hz ASSR in healthy adult males using graph theory. The second objective was to identify a population in which tDCS effectively modulates the brain network of 40 Hz ASSR.

**Methods:**

This study used a randomized, sham-controlled, double-blinded crossover approach. Twenty-five adult males (20–24 years old) completed two sessions at least 1 month apart. The participants underwent cathodal or sham tDCS of the dorsolateral prefrontal cortex, after which 40 Hz ASSR was measured using magnetoencephalography. After the signal sources were mapped onto the Desikan–Killiany brain atlas, the statistical relationships between localized activities were evaluated in terms of the debiased weighted phase lag index (dbWPLI). Weighted and undirected graphs were constructed for the tDCS and sham conditions based on the dbWPLI. Weighted characteristic path lengths and clustering coefficients were then measured and compared between the tDCS and sham conditions using mixed linear models.

**Results:**

The characteristic path length was significantly lower post-tDCS simulation (*p* = 0.04) than after sham stimulation. This indicates that after tDCS simulation, the whole-brain networks of 40 Hz ASSR show a significant functional integration. Simple linear regression showed a higher characteristic path length at baseline, which was associated with a larger reduction in characteristic path length after tDCS. Hence, a pronounced effect of tDCS is expected for those who have a less functionally integrated network of 40 Hz ASSR.

**Discussion:**

Given that the healthy brain is functionally integrated, we conclude that tDCS could effectively normalize less functionally integrated brain networks rather than enhance functional integration.

## 1. Introduction

Transcranial Direct Current Stimulation (tDCS) is a neuromodulation technique that modifies cortical excitability by applying a weak direct current. According to pharmacological studies, the immediate and short-term effects of tDCS are caused by the polarity-specific modulation of the neuronal membrane potential. Anodal tDCS depolarizes the resting membrane potential and increases the spontaneous firing rate, whereas cathodal tDCS has the opposite effect, leading to hyperpolarization and a reduced firing rate (1, 2). If tDCS is applied for an increased duration, such as up to several minutes or even longer, the changes in excitability persist for over an hour after the stimulation has ceased. This is referred to as the “after-effect.” The after-effect is not solely dependent on the modulation of the neuronal membrane potential but is prevented by N-methyl-D-aspartate (NMDA) receptor antagonists (3), prolonged by NMDA agonists (3), and enhanced by a GABA receptor agonist (4), possibly reflecting GABAergic modulation of neuroplastic changes [e.g., long-term potentiation (5)]. These findings suggest that the after-effects of tDCS may be caused by alterations in the activity of NMDA receptor-positive GABAergic interneurons.

tDCS has been shown to modulate behavior and cognitive function, stimulate the dorsolateral prefrontal cortex (DLPFC), and enhance attention control and executive functions (6, 7). As a result, tDCS has shown promising effects in treating neurological and psychiatric conditions such as depression, dementia, and schizophrenia (8). Historically, electrode placement for tDCS has relied on the assumption that specific brain regions correspond to distinct functions and that complex cognitive processes are mediated by functionally independent areas. However, recent evidence challenge this notion, and suggest that cognitive function is governed by dispersed regions in the brain acting together (9, 10). Consequently, stimulating a particular brain region with tDCS may not exclusively modulate a single cognitive function but could affect multiple functions, thereby, influencing larger cortical areas by altering the excitability of all neurons within it. Furthermore, studies have found that tDCS-induced polarity-specific effects are not limited to the stimulated sites (11–13). For instance, Pellegrino et al. (13) showed that tDCS at the primary sensory motor hand region inhibited an auditory evoked potential in distant regions. Moreover, two other studies reported altered regional brain connectivity, both near the primary stimulation site and in the associated regions (11, 12). Therefore, tDCS-induced cortical modulation should be considered a complex interplay of excitation and inhibition across distributed regions of the brain (14). Given that cognitive function is governed by distributed brain regions acting in parallel and that tDCS-induced polarity-specific effects extend from the stimulated sites to adjacent or even distant regions, solely measuring the activity or connectivity between two specific brain regions would oversimplify the situation. To gain a deeper understanding of the neuronal mechanisms underlying tDCS effects on the stimulation area, it is crucial to broaden our scope and understand its influence on larger brain networks.

ASSR is an oscillatory event-related potential that continuously phase-locks to the frequency of a given auditory stimulus over a period of stimulation (15), reflecting the ability of the sensory cortex to respond to a modality-specific stimulus (16). In humans, ASSR peaks when the auditory stimulus is delivered at 40 Hz (17). The neural underpinnings of the 40 Hz ASSR are believed to involve circuits composed of fast-spiking parvalbumin-positive (FSPV) GABAergic interneurons and pyramidal cells within the sensory cortex (18–21). The activation of NMDA receptors on FSPV interneurons is crucial for generating 40 Hz entrainment (20, 21). In line with these findings, both *in vitro* and *in vivo* studies have demonstrated that NMDA receptor agonists can either disrupt (22) or potentiate (23) gamma oscillations of approximately 40 Hz (i.e., 30–80 Hz). Although the 40 Hz ASSR in response to an auditory stimulus has been considered to be localized to the primary auditory cortex [i.e., the superior temporal plane (24, 25) or Heschl’s gyrus (26)], recent research indicates that the sources of the 40 Hz ASSR extend beyond the auditory cortex (27–29). It has been observed in frontal and subcortical regions (28), occipital lobe, precentral gyrus, superior parietal lobe (27), as well as parietal and frontal areas (29). Moreover, the ASSR in these regions is not mutually exclusive; rather, these cell clusters seem to interact in an organized fashion (30). This evidence suggests that a 40 Hz ASSR can be generated by a network of widely distributed brain regions.

Griskova-Bulanova and colleagues recently reviewed the current state-of-art knowledge on how various non-invasive brain stimulation techniques (NIBS), such as transcranial alternating current stimulation (tACS), tDCS, transcranial random noise stimulation (tRNS), paired associative stimulation (PAS), and repetitive transcranial magnetic stimulation (rTMS), affect gamma-range ASSRs in both healthy and clinical populations (31–34). It was found that the research so far has been inconsistent and methodologically heterogeneous, with evidence showing that NIBS techniques can enhance, decrease, or have no effect on ASSRs. As a result, they emphasized the need to further explore the mechanisms underlying the modulation of gamma-range ASSRs by NIBS. Among NIBS techniques, tDCS is of particular interest due to its activation of NMDA receptors on FSPV GABAergic interneurons, which play a crucial role in generating the 40 Hz ASSR. However, the evidence is inconsistent. A recent pre-print study by Marshall (35) showed that tDCS alters the waveform of 40 Hz oscillations in healthy adults, while Pellegrino et al. reported a significant effect of tDCS (anode on C3, cathode on C4) on inter-trial phase coherence (ITPC) and power of individual sources of ASSR in healthy individuals (13). On the contrary, Ahn et al. applied tDCS (anode between F3 and Fp1, cathode between T3 and P3) and found no significant effect on the amplitude and ITPC of ASSR (36). Similarly, Miyagishi et al. used tDCS (anode on F3, cathode on F4) on healthy adults and observed no significant impact on ITPC and event-related spectral perturbation (37). Notably, these studies focused on individual brain regions; however, as tDCS affects large areas of the cortex, its global modulation impact should be considered rather than a local change in these interneurons. Hence, studies focusing on individual brain regions may not fully capture the effect of tDCS. Therefore, investigating the effect of tDCS on 40 Hz ASSR at the neural network level could provide a more comprehensive understanding of its broader influence on interconnected brain regions.

With the help of graph theory, we would be able to explore the effect of tDCS on the network of 40 Hz ASSR. In electroencephalography or magnetoencephalography (MEG) studies, the brain can be divided into discrete regions that mutually interact over the course of time. To understand the properties of this complex network, neuroscientists use graph theory (38). Graph theory essentially reduces complex systems to just a “graph,” a set of nodes connected by edges. Particularly, a neural network is defined as a graph, where the nodes represent distinct brain regions and the edges represent connectivity between any two brain regions (39). Graph theory provides measures for describing the characteristics of graphs as single numerical values. Among the various theoretical measures of graphs, the mean clustering coefficient (C) and average shortest path length (L) are simple, well-established, and widely used. C represents the degree to which the connected nodes form local clusters and higher C is held to correspond to the brains’ tendencies to process information locally (i.e., functional segregation) (40). L represents the average number of edges to be crossed from one node to another, where the average is taken over all possible pairs of nodes. Thus, a shorter L corresponds to the network’s tendency to integrate information from remote brain regions (i.e., functional integration) (40). Structural and functional studies have suggested that healthy human brain networks attain both high C and short L, which lie between an ordered (high C and long L) and a randomly generated graph (low C and short L) (41), suggesting an optimal balance between functional integration and segregation (41). However, it is clear that this balance is altered in some brain disorders, such as depression (42), dementia (43), and schizophrenia (44). It is noteworthy that, as mentioned earlier, tDCS has been proven to be beneficial in treating these disorders. Taken together, this implies that tDCS may normalize the deviated balance of the brain network in these disorders.

tDCS can target various brain regions, but in recent years, DLPFC has garnered increasing interest for its therapeutic potential in neurological and psychiatric disorders. Li et al. (45) demonstrated that tDCS targeting the DLPFC is promising to treat neuropsychiatric disorders. There are several electrode placement options when applying tDCS to the DLPFC. As described by Li et al. (45), most researchers place the anode on the left DLPFC (F3) and the cathode on the contralateral cortex. Among these configurations, bilateral DLPFC with the cathode on the right DLPFC (F4) is one of the most common montages. This tDCS montage has proven effective in treating depression (46–48), schizophrenia (49), addiction (50, 51), ADHD (52), and anxiety (53). Notably, evidence suggests that alterations in FSPV interneurons may play a crucial role in the pathophysiology of these disorders (54–56). Given the therapeutic effects of tDCS on these disorders and the impact of tDCS on NMDA-positive interneurons, it is reasonable to infer that tDCS may exert its therapeutic effects by regulating the activity of the FSPV interneurons.

In this study, we aimed to investigate the impact of tDCS on the 40 Hz ASSR network by utilizing graph theory. Building upon the findings of Ying et al. (30), which examined the ASSR network in patients with schizophrenia and reported a significantly increased L compared to healthy controls. Hence, considering the potential of tDCS to normalize aberrant networks in psychiatric disorders, we hypothesized that tDCS could decrease L in the 40 Hz ASSR network, even in healthy individuals. Additionally, we comprehensively analyzed the effect of tDCS on C under similar conditions. Another objective of this study was to explore the relationship between the properties of the intrinsic 40 Hz ASSR network and the reduction in L.

## 2. Materials and methods

### 2.1. Experimental design

**Figure 1:**
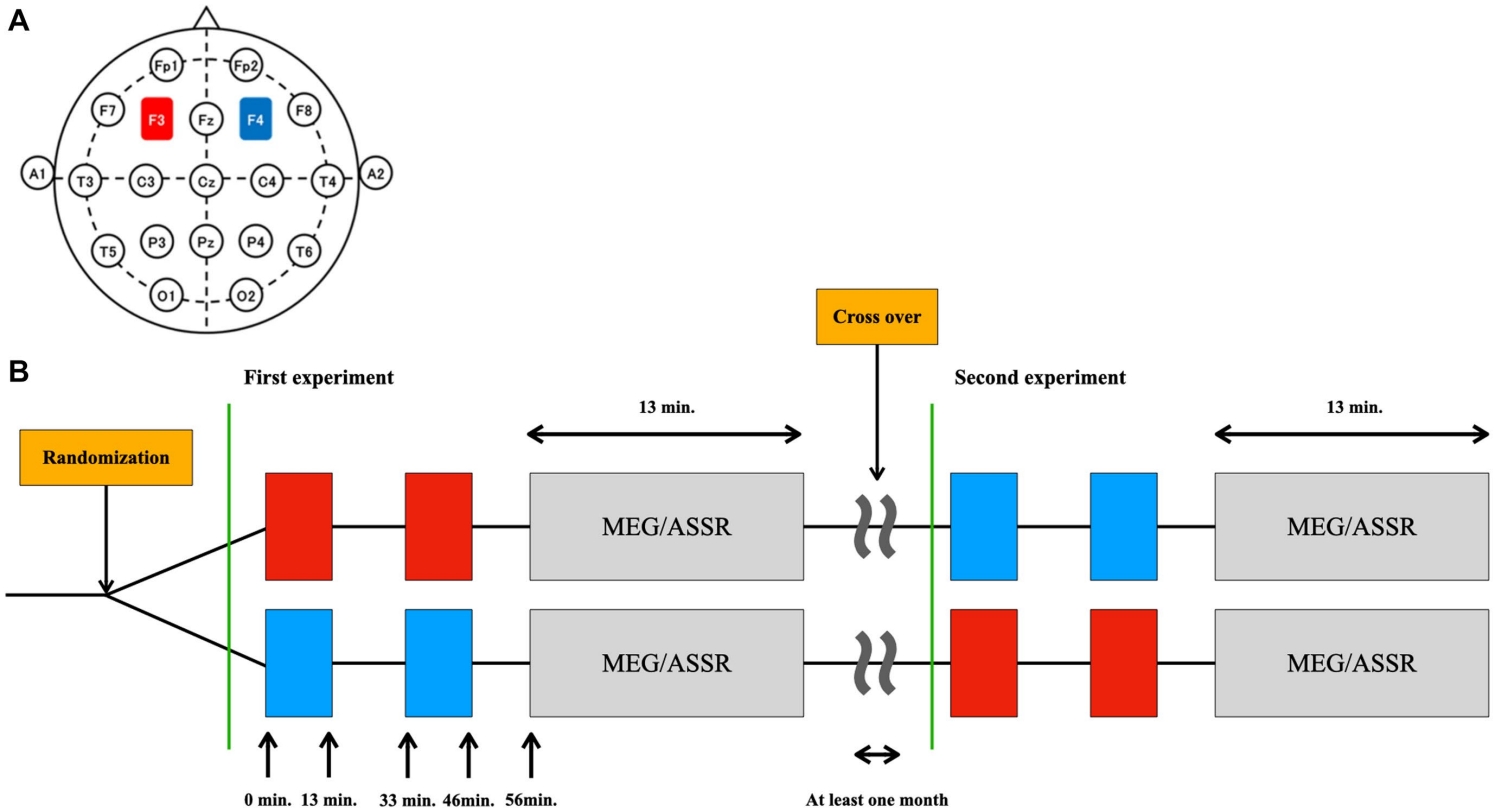
Design of the study. **(A)** The placement of the electrodes was based on the international 10–20 EEG system: the anode (red) at the F3 (left DLPFC) and the cathode (blue) at the F4 (right DLPFC). **(B)** The type of stimulation (tDCS or sham) was randomized in a double-blinded manner. Thirteen minutes of tDCS or sham was applied twice with an interval of 20 min. ASSR was measured using MEG 10 min after the cessation of stimulation (at 56 min after initiation of the stimulation). This MEG/ASSR session takes 13 min in total. The participants underwent the simulation experiment twice in a crossover fashion at least one month apart.

This study used a randomized, sham-controlled, double-blinded crossover design. Participants completed two sessions at least 1 month apart to control for carryover effects. The participants underwent 26 min of cathodal or sham tDCS of the DLPFC, after which ASSR was measured (Figure 1).

The study design primarily focused on measuring the after-effects of tDCS and did not include assessments of the brain’s state before stimulation. However, previous studies have demonstrated that the effects of brain stimulation can be significantly influenced by the brain’s state at the time of stimulation (57, 58). The concept of state dependency is essential for interpreting tDCS outcomes, as it highlights that the effects of an external stimulus depend not only on the stimulus properties but also on the susceptibility of the stimulated brain region to activation (59, 60). According to Zagha and McCormick (61), a brain state is “a recurring set of neural conditions that is stable for a behaviorally significant period of time.” It is important to consider that brain states can fluctuate over various timescales, such as years (e.g., developmental maturation, aging), hours (e.g., sleep stages, mood), or even hundreds of milliseconds (e.g., attention shifts). In this context, it has been shown that the effects of brain stimulation can be significantly influenced by these fluctuating brain states. For example, the impact of brain stimulation on working memory varies with the subject’s age (62) and its effect on mood depends on the subject’s arousal state (63); moreover, attention shifts can also affect the stimulation outcomes (64). In this study, we accounted for brain state fluctuations occurring over months, but we were unable to consider shorter fluctuations that take place within hours or seconds. Hence, it is essential to acknowledge this limitation when interpreting the findings of this study.

### 2.2. Participants

Twenty-five right-handed adult, native Japanese-speaking males (mean age = 21.4, range = 20–24 years) from Kanazawa University were included in the study. The exclusion criteria were as follows: (i) deafness, (ii) self/family history of any neuropsychiatric disorder, and (iii) the use of any ongoing medication. The complete IQ score was estimated using the Japanese version of the National Adult Reading Test [mean = 108.9, range 93–122, (65)]. One participant completed the MEG recordings for both the sham and tDCS conditions; however, the tDCS condition recording was contaminated by excessive noise. This participant was excluded from the analysis. In addition, two participants were excluded from the statistical analysis: one due to drowsiness during the MEG recording, and the other due to noise from a tooth filling. Consequently, our analysis included 22 right-handed adult males. Participants’ characteristics are presented in Table 1. Written informed consent was obtained from each participant prior to their participation. The Ethics Committee of Kanazawa University Hospital approved the study’s methods and procedures, which were conducted in accordance with the Declaration of Helsinki. The participants in this current study overlap with those of our previous research (37); however, none of the results presented here overlap with the findings of the earlier study (37). Furthermore, the focus and objectives of the earlier study were distinct from those of the current investigation.

**Table 1:** Characteristics of participants.

	Participants
*N*	22
Age	21.4 (1.3)
IQ^†^	107.7 (5.6)
Education years	14.9 (1.3)

Numbers are mean (standard deviation) or counts.

^†^IQ was estimated using the Japanese version of the National Adult Reading Test.

### 2.3. Intervention: tDCS

The settings for tDCS or sham stimulation were the same as those used in our previous studies (37, 66, 67). Briefly, direct current was delivered through two saline-soaked electrodes (35 cm^2^) using a stimulator (DC-STIMULATOR Plus; NeuroConn GmbH, Germany). The anode was placed over the left DLPFC (F3) and the cathode was placed over the right DLPFC (F4) according to the international 10–20 EEG system (Figure 1A). The order of stimulation type (tDCS or sham) was randomized and counterbalanced across participants: 11 completed sham followed by tDCS simulation, and the remaining 11 completed tDCS followed by sham (Figure 1B).

In our study, we delivered tDCS before the ASSR measurements (Figure 1B). Participants received two 13-min stimulation sessions separated by a 20-min inter-stimulation interval, following the protocol used by Miyagishi et al. (37). During the sham stimulation, participants were stimulated for only the first 10 sec for both the blocks, which prevented the participants from noticing the absence of electrical stimulation. While we did not explicitly require participants to report any perceived sensations of the active and sham tDCS sessions, some of them instantaneously mentioned the initial tingling sensation. Importantly, none of the participants reported being able to differentiate between the active tDCS and sham stimulation conditions, suggesting that the blinding procedures were effective.

We implemented the 20-min interstimulus interval with no stimulation for tDCS administration based on the findings of Monte-Silva et al. (68). Their research demonstrated that periodic anodal tDCS can induce long-lasting LTP-like excitability enhancements. They found that when the second stimulation was applied during the after-effects of the first one (with an interval of 3 or 20 min), the combined after-effects of both blocks persisted for over 24 h post-tDCS. In contrast, intervals of 3 or 24 h led to the abolishment of tDCS after-effects, suggesting that the excitability changes achieved through spaced tDCS are not solely attributable to the total stimulation duration. These LTP-like excitability enhancements were found to be NMDA receptor-dependent, as evidenced by the fact that an NMDA receptor antagonist blocked them. We selected the 20-min interstimulus interval approach for two main reasons: firstly, because the after-effects are long enough to be recorded using MEG upon stimulation before the ASSR measurements [i.e., at least several hours post-stimulation (69)]; and secondly, due to the effects being NMDA receptor-dependent, which implies a direct link to the neural mechanisms underlying tDCS’s impact.

Here, ASSR was measured using MEG 10 min after ceasing the stimulation (at 56 min after initiation of the stimulation). This MEG/ASSR session takes 13 min in total. The participants underwent the stimulation experiment twice in a crossover fashion, with at least 1 month apart between sessions.

### 2.4. Magnetoencephalography and MRI recordings

The MEG and MRI recordings were performed similarly to our previous study (37). Magnetic fields were recorded with a whole-head-type MEG system (MEGvision PQA160C; Ricoh Company Ltd., Kanazawa, Japan), which featured 160 channels configured as first-order coaxial gradiometers with a baseline of 50 mm. Each coil of the gradiometers was 15.5 mm in diameter. The MEG signals were sampled at 2000 Hz per channel using a 500 Hz low-pass filter.

For MRI recordings, we used a Signa Excite HD 1.5 T system (GE Yokogawa Medical Systems Ltd., Milwaukee, WI, United States) to obtain T1-weighted structural images with spherical lipid markers (observed as high-intensity legion) placed at the five MEG fiduciary points. The T1-weighted image consisted of 166 sequential 1.2 mm-thick slices with a resolution of 512 × 512 points within a field of view of 261 × 261 mm. We reconstructed the cortical surface using the FreeSurfer software (version 5.3^1^).

To co-register the MEG and MRI images for each participant, we merged them based on the location of the markers.

### 2.5. Auditory steady state response

The procedure for the ASSR session was identical to our previous study (37). During each MEG recording, participants were instructed to look at a white fixation cross on a black background presented on a screen in front of them. The participants were given auditory stimulation to induce ASSR. Auditory stimulation consisted of 250 trials of click-train stimuli. The stimuli were presented binaurally at 80 dB for 1,000 ms each, with inter-trial intervals of 2000 ms. Each click-train stimulus was a series of 1 kHz single sine-wave stimuli administered at a stimulation frequency of 40 Hz. Stimuli were received using nonmagnetic stereo earphones with earplugs (ER-30; Etymotic Research Inc., IL, United States). This MEG/ASSR session lasted 13 min in total.

The participants were required to perform one task to stay awake which was to detect the 2 kHz click-train stimulus presented 10 times (i.e., 4% of the total stimulus) during the session. When the 2 kHz stimulus was given, the participants pressed a button (LUMINA LU400-PAIR, Cedrus Corporation, CA, United States) with their right index finger. All stimuli were controlled using Presentation software (version 13.1; Neurobehavioral Systems, CA, United States) for Windows XP.

### 2.6. Magnetoencephalography preprocessing

All data processing and analytical procedures were performed using Brainstorm (70) and FreeSurfer (71), with additional scripts developed in MATLAB R2021a (MathWorks, Natick, MA, United States). We preprocessed the MEG data following the procedures outlined in the Brainstorm tutorial.^2^ Initially, noisy sensors were identified through visual inspection and excluded from the analysis. Depending on the sensor conditions on the day of the examination, up to three sensors were removed on a case-by-case basis. Subsequently, eye movement and cardiac artifacts were removed using the signal-space projection method. Segments containing head movements or muscle artifacts were discarded by visual inspection or automatic processing in Brainstorm.

### 2.7. Co-registration of MEG on MRI image

We co-registered the MEG recordings on individual MRI images according to the marker locations. Five markers were recorded using MEG and MRI: the nasion, midline frontal, vertex, and bilateral mastoid processes. We used five coils to generate a magnetic field for MEG. For the MRI, we used five pieces of lipid capsules, which were observed as high-intensity regions. Volumetric segmentation was performed using FreeSurfer software.

### 2.8. Source reconstruction and segmentation

The head model was computed using an overlapping sphere algorithm (72) with a lower-resolution cortical surface representation of 15,000 vertices. The inverse solution was calculated for each using weighted minimum-norm estimation with standardized low-resolution brain electromagnetic tomography (sLORETA) (73, 74). A noise covariance matrix was computed using the MEG recordings captured during the −500 to 0 ms time window for each epoch within a session. To account for potential slow shifts in the data, we removed the DC offset block-wise, subtracting the average value of each channel from its respective block before concatenation. Subsequently, these pre-stimulation baseline segments from individual trials were concatenated to create a comprehensive matrix, from which the sample noise covariance was calculated. We grouped the sources into 68 regions of interest according to the Desikan–Killiany atlases (75) using principal component analysis. The epochs were then defined as −500 to 1,000 ms in relation to the auditory stimulus onset (0 ms). After segmentation, the data were then baseline-corrected with respect to the mean of the pre-stimulus period (from −500 to 0 ms). Specifically, we calculated the average value for each channel in the baseline (i.e., pre-stimulus period) and then subtracted it from the channel at all time instants over the entire epoch interval (from −500 to 1,000 ms).

### 2.9. Time-frequency analysis

Previously, we conducted a time-frequency analysis and the results of which were published in Miyagishi (37). The analysis employed Morlet wavelets with a central frequency of 2 Hz, while the mother wavelet featured a time resolution of 3 s. We calculated event-related spectral perturbation (ERSP) and inter-trial phase coherence (ITPC) values within a frequency range of 2 to 50 Hz. Our time-frequency maps disclosed a prominent ERSP at approximately 40 Hz across various temporal lobe regions, including the entorhinal, middle temporal, inferior temporal, fusiform, parahippocampal, superior temporal, temporal pole, and transverse temporal areas. Furthermore, the ERSP extended into some regions of the occipital (lateral occipital), frontal (lateral orbitofrontal, medial orbitofrontal, and precentral), and parietal (postcentral and supramarginal) lobes.

Similarly, ITPC values peaked around 40 Hz in many areas within the temporal lobe, as well as the frontal (caudal middle frontal, lateral orbitofrontal, medial orbitofrontal, parsopercularis, parsorbitalis, parstriangularis, precentral, and rostral anterior cingulate areas), occipital (cuneus and lateral occipital), and parietal lobes (inferior parietal, postcentral, posterior cingulate, supramarginal, superior parietal regions, and precuneus areas).

These time-frequency maps for all ROIs can be found in the supporting information in Supplementary Figure S1 (ERSP) and Supplementary Figure S2 (ITPC) of the published manuscript (37). These observations from our previous study corroborate the more recent perspective that the sources of the 40 Hz ASSR extend beyond the auditory cortex, encompassing frontal, occipital, parietal, and subcortical regions (27–29).

### 2.10. Graph construction based on dbWPLI

A graph serves as a topographical representation of a network, consisting of “nodes” and “edges” that connect pairs of nodes (39, 40). In this study, the nodes corresponded to 68 brain regions of interest from the Desikan–Killiany atlases (75). The edges, which represent the functional connectivity between pairs of brain regions, were weighed using the corresponding values of the dbWPLI (76). We utilized the absolute values of the dbWPLI, following Kuntzelman’s approach (77). This method offers significant advantages over other phase-lagged connectivity indices, such as minimal sampling bias and enhanced ability to detect true phase synchronization (76, 77). The absolute values of the dbWPLI range from zero to one, with larger values indicating stronger functional connectivity. A detailed description of the dbWPLI is provided in the Supplementary material.

Previously (37), under the same stimulation protocol and MEG recording conditions, a prominent ASSR peak was observed around 38–42 Hz in numerous regions. However, the effect extended to an adjacent frequency range from 32 Hz to 48 Hz. Thus, in this study, we chose the 32–48 Hz frequency range with a 2 Hz margin (i.e., 30–50 Hz in total) to capture the entire 40 Hz ASSR brain network.

For each participant, we computed the dbWPLI by applying a combination of Tukey and Parzen windows, following the connectivity toolbox of Brainstorm. Fast Fourier transforms (FFT) was computed for every 1,000 ms segment (i.e., the period of the auditory stimulus) for each of the 68 estimated signal sources. From the cross spectra between each pair of sources, dbWPLI were calculated (76) (refer to Supplementary materials for details). The dbWPLI values were averaged over 30–50 Hz. Subsequently, the absolute value of the dbWPLI was averaged across all epochs, and this value was used as the corresponding edge weight. As a result, we constructed a dbWPLI-based undirected weighted functional connectivity matrix (68 × 68) for each participant under both experimental conditions (tDCS and sham). We analyzed weighted networks rather than binary networks because thresholds for binary matrices are often arbitrarily chosen, and we believe that weak connections could also provide information about the network (52).

### 2.11. Graph metrics

The two most commonly used characteristics for weighted networks, the characteristic path length (L^w^) and average clustering coefficient (C^w^) (40) were calculated using MATLAB functions and the Brain Connectivity Toolbox (BCT, available at^3^).

L^w^ measures functional integration in the brain (i.e., the ability to rapidly combine specialized information from distributed brain regions), based on the concept of paths and their lengths. In a weighted undirected graph, a path denotes a sequence of distinct nodes connected by connected edges. The length of a path is the smallest sum of the inverse weights of the edges, with a shorter path length implying a stronger potential for integration. Technically, L^w^ is defined as the average shortest path length for all the possible paths in the network.


$$L^{w}\equiv \frac{1}{N}\underset{\left\{j\in N\right\}}{\sum }\frac{\sum_{j\neq i}d_{ij}^{w}}{N-1}$$

where N is the number of nodes and dij is the shortest weighted path length between the i^th^ and j^th^ node (78).

C^w^ measures functional segregation (i.e., the ability of specialized processing to occur within densely interconnected groups of brain regions). C^w^ primarily quantifies the presence of groups known as “clusters” in the network. The presence of clusters in functional brain networks suggests the organization of segregated information processing (40). C^w^ was calculated by averaging the weighted clustering coefficient for each node and then averaging across all 68 nodes after rescaling the connection weight. We omitted the technical and mathematical definitions of C^w^ [a formal mathematical definition is presented elsewhere (79)].

In our analysis, we initially calculated L^w^ and C^w^ for each brain lobe to evaluate the local effects of tDCS. In particular, according to the Desikan-Killiany brain atlas, we separated estimated sources into right or left frontal (superior frontal, rostral and caudal middle frontal, pars opercularis, pars triangular, pars orbital, lateral and medial orbitofrontal, precentral, paracentral, and frontal pole), parietal (superior parietal, inferior parietal, supramarginal, postcentral, precuneus), temporal (superior, middle, and inferior temporal, banks of the superior temporal sulcus, fusiform, transverse temporal, entorhinal, temporal pole, and parahippocampal) and occipital (lateral occipital, lingual, cuneus, pericalcarine) lobes (75, 80). Then we constructed a “sub-graph” within each region. The nodes of the subgraphs correspond to nested brain regions; for example, nodes of subgraphs within the frontal lobe include the superior frontal, rostral and caudal middle frontal, pars opercularis, pars triangular, pars orbital, lateral and medial orbitofrontal, precentral, paracentral, and frontal poles. The edges of these subgraphs were similarly defined in terms of the dbWPLI.

### 2.12. Statistical analysis

Our main objective was to investigate the effect of tDCS (in relation to sham stimulation) on L^w^, which represents functional integration in the ASSR network, but for totality, we also analyzed the effect of tDCS on C^w^, which represents functional segregation in the ASSR brain network. Another aim was to examine whether the effect of tDCS was dependent on L^w^ after sham stimulation.

First, we performed separate linear mixed-effect analyzes for network-level L^w^ and C^w^, based on the type of stimulation (tDCS vs. sham). We included random intercepts for each subject to account for individual variability. Since we designated the analysis for L^w^ as preplanned and treated the C^w^ analysis as exploratory, we did not apply any correction for multiple comparisons between these two tests (81). Another reason we did not perform a correction for multiple comparisons was because L^w^ and C^w^, although quantifying different aspects of the graph structure, might not be strictly mutually independent (81). For instance, a graph with densely clustered nodes might have a high-weighted clustering coefficient and a relatively low-weighted characteristic path length, as these clusters facilitate shorter paths between nodes.

Second, we explored the “local” effects of tDCS (in relation to sham) on L^w^ and C^w^ in each subgraph for the regions of interest (i.e., right or left frontal, parietal, temporal, and occipital lobes). This resulted in distinct linear mixed-effect models for every region. These models included the type of stimulation as a within-subjects fixed effect and subjects as a random effect, allowing for random intercepts for each subject. Given the exploratory nature of this analysis, we conducted 16 statistical tests without adjusting for multiple comparisons (82).

Next, we examined whether the effect of tDCS was dependent on the value of L^w^ after the sham condition, for which we performed a simple linear regression. In this model, we calculated the difference between stimulation conditions (i.e., after tDCS – after sham) and used the after-sham L^w^ values to predict this difference.

Before we applied linear regression models, we used standard methods to verify that our data met the assumptions for regression analysis (e.g., normality of the overall error distribution, homogeneity of variance, etc.). Visual inspection of residual plots did not reveal any obvious deviations from normality, but the assumption of homogeneity was violated in some models. For those models, we, therefore, used heteroscedasticity-robust standard errors (83). Statistical significance was inferred for *p* < 0.05, and *p* < 0.025 for posthoc analysis. All statistical analyzes were conducted using Stata software (Stata ver. 15.0; Stata Corp., College Station, TX, United States).

## 3. Results

### 3.1. Effect of tDCS on L^w^ or C^w^ of the network of ASSR

**Figure 2:**
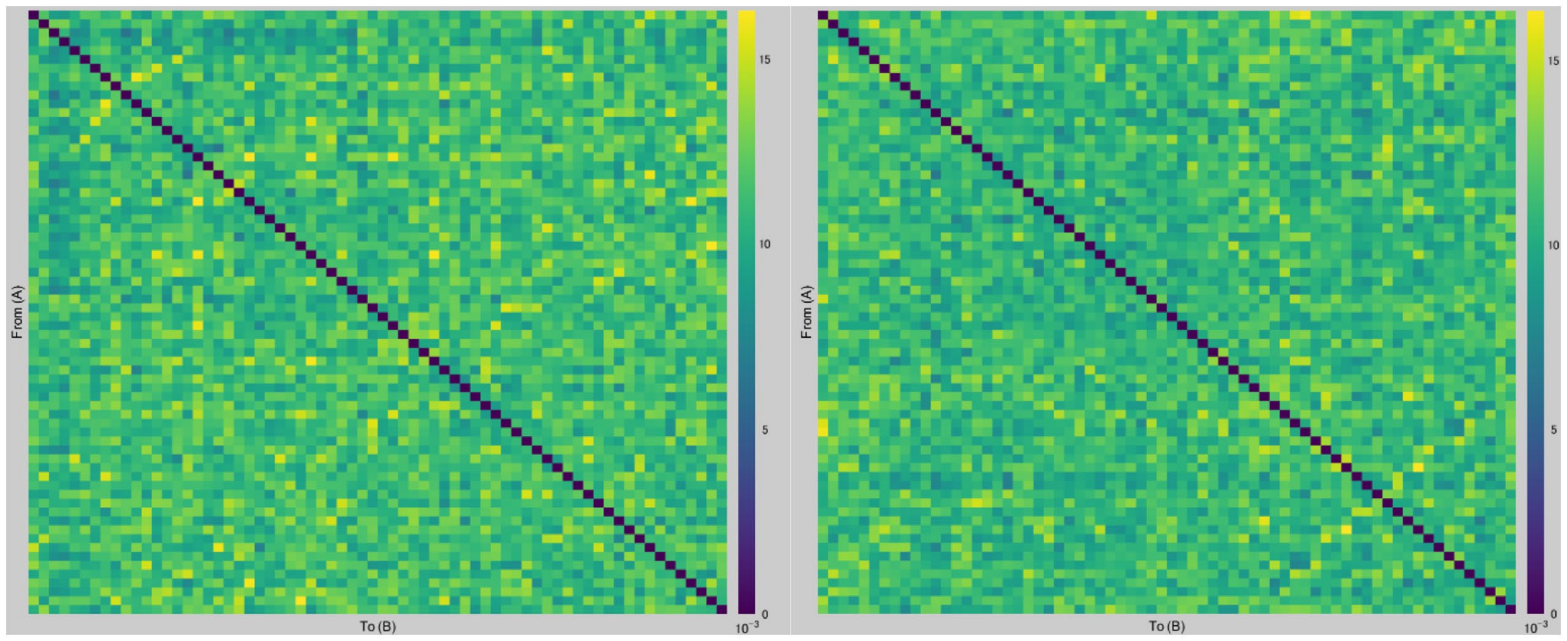
Examples of dbWPLI-based undirected weighted functional connectivity matrices. The figure displays representative graphs of dbWPLI of a subject, which were used to calculate the weighted characteristic path length and weighted clustering coefficient. Rows and columns represent corresponding nodes (i.e., regions of interest in the Desikan-Killiany atlas). For instance, a number in row 12 and column 66 corresponds to the dbWPLI value between the right frontal pole (region 12) and the right temporal pole (region 66). The left figure was constructed using data from the after-sham condition, while the right figure was constructed using data from the after-tDCS condition.

Figure 2 presents a graph of dbWPLI-based undirected weighted functional connectivity matrix (68 × 68) of a representative subject.

**Figure 3:**
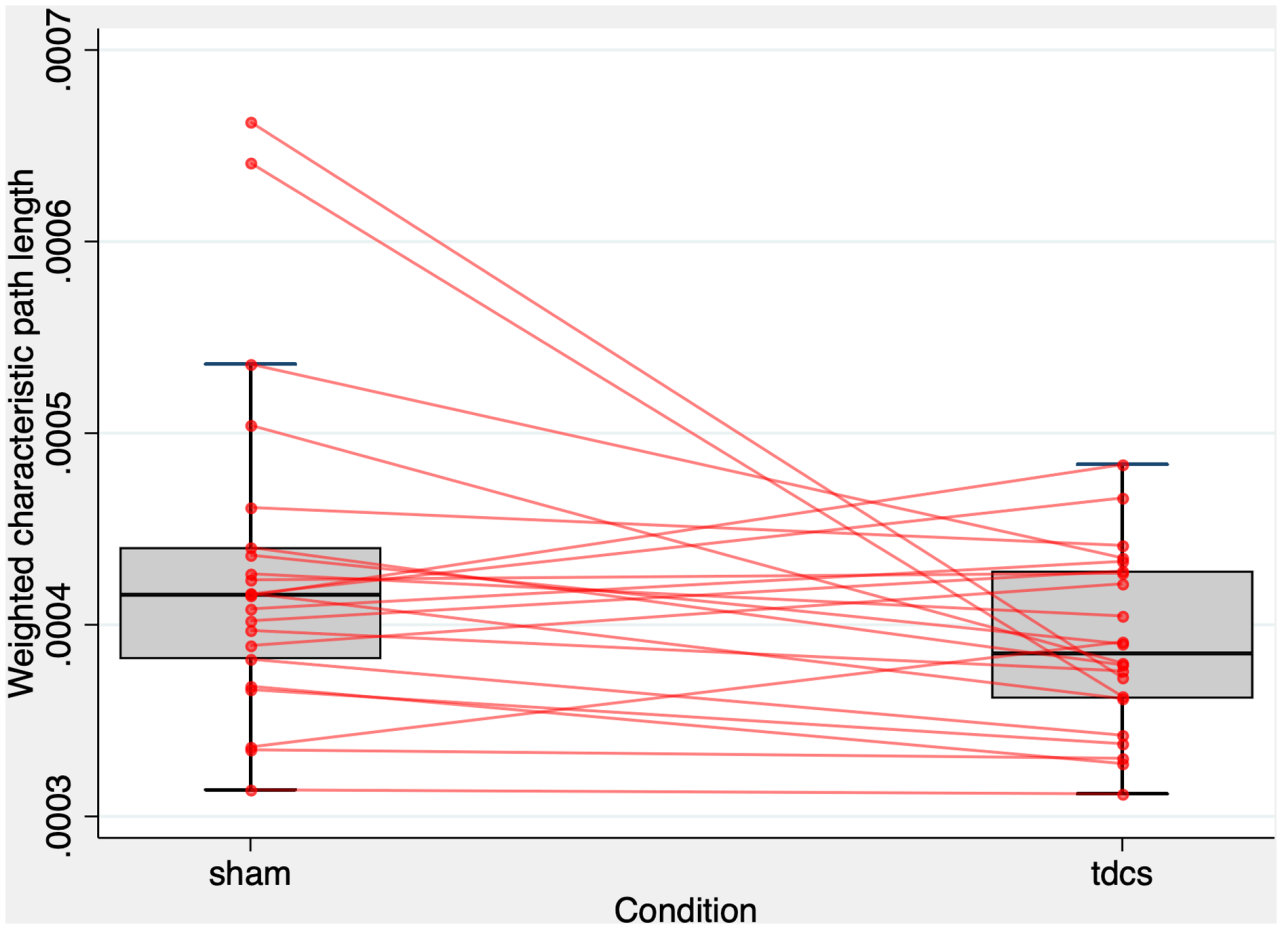
L^w^ of the whole-brain network after tDCS and sham condition. The figure displays a combined scatter plot and box plot of weighted characteristic path length (L^w^) values across two conditions: sham and tDCS. The box plot elements comprise the first quartile, median, and third quartile values, represented by horizontal bars. Vertical lines (whiskers) extend from the first and third quartiles to the minimum and maximum values within 1.5 times the interquartile range, illustrating the range of the data. Additionally, small red circles represent individual data points for each subject in the two conditions. The horizontal-axis indicates the experimental condition, while the vertical-axis represents the L^w^ values. The figure aims to provide a visual representation of the distribution and spread of L^w^ values in both experimental conditions.

We developed a model that predicted the L^w^ of the whole brain network (22 subjects, 44 observations) based on the types of stimulation (tDCS vs. sham) and random intercepts for subjects and found that the types of stimulation were significant predictors of L^w^ (z = −2.02, *p* = 0.044). The estimated standard deviation of the random intercepts was 0.26 × 10^−4^, with a robust standard error of 0.14 × 10^−4^. Figure 3 shows the patterns of L^w^ in the whole-brain network. The effect of the types of stimulation was non-significant to predict C^w^ (z = −1.55, *p* = 0.122). In contrast, the models predicting the L^w^ or C^w^ of each sub-graph (i.e., right or left frontal, temporal, parietal, and occipital) did not show any significant effect (Supplementary Tables S1, S2).

As illustrated in Figure 3, only two subjects exhibited much smaller L^w^ values after tDCS compared to the sham condition. To address concerns that the significant effect of tDCS on L^w^ might be driven by these potential outliers showing extreme changes, we calculated the difference in L^w^ between the sham and tDCS conditions (i.e., L^w^ after tDCS - L^w^ after sham) and evaluated the z-scores for these two subjects. Their z-scores were 2.58 and 2.71, respectively, falling below the standard cut-off value that identifies outliers (84). Additionally, we employed the interquartile range (IQR) method to establish outlier fences. The first (Q1) and third quartiles (Q3) were at −0.24 × 10^−4^ and 0.55 × 10^−4^, respectively. Consequently, the IQR was calculated as 0.79 × 10^−4^. As a result, these two subjects fell within the range of the upper inner and upper outer fences (i.e., between Q3 + 1.5 IQR and Q3 + 3IQR). These findings suggest that the two subjects were mild outliers (84). To further address this concern, we performed a one-tailed Wilcoxon rank test to determine whether L^w^ was larger in the sham condition than in the tDCS condition. We chose a one-tailed test based on our hypothesis, which was informed by the results of a linear mixed-effect analysis and visual inspection of a scatter plot (Figure 3), predicting that tDCS could lead to the reduction of L^w^. The one-tailed test offers greater statistical power than a two-tailed test at the same significance level. The Wilcoxon rank test revealed that L^w^ was significantly higher in the sham condition compared to the tDCS condition (z = 1.704, Prob > z = 0.044).

Altogether, these results indicate that the whole-brain network of the ASSR becomes functionally more integrated after tDCS compared to sham stimulation. However, tDCS-induced changes in functional segregation and its “local” effects on functional integration were not significant in any brain regions.

### 3.2. L^w^ at baseline and effect of tDCS on L^w^ of the whole-brain network of ASSR

**Figure 4:**
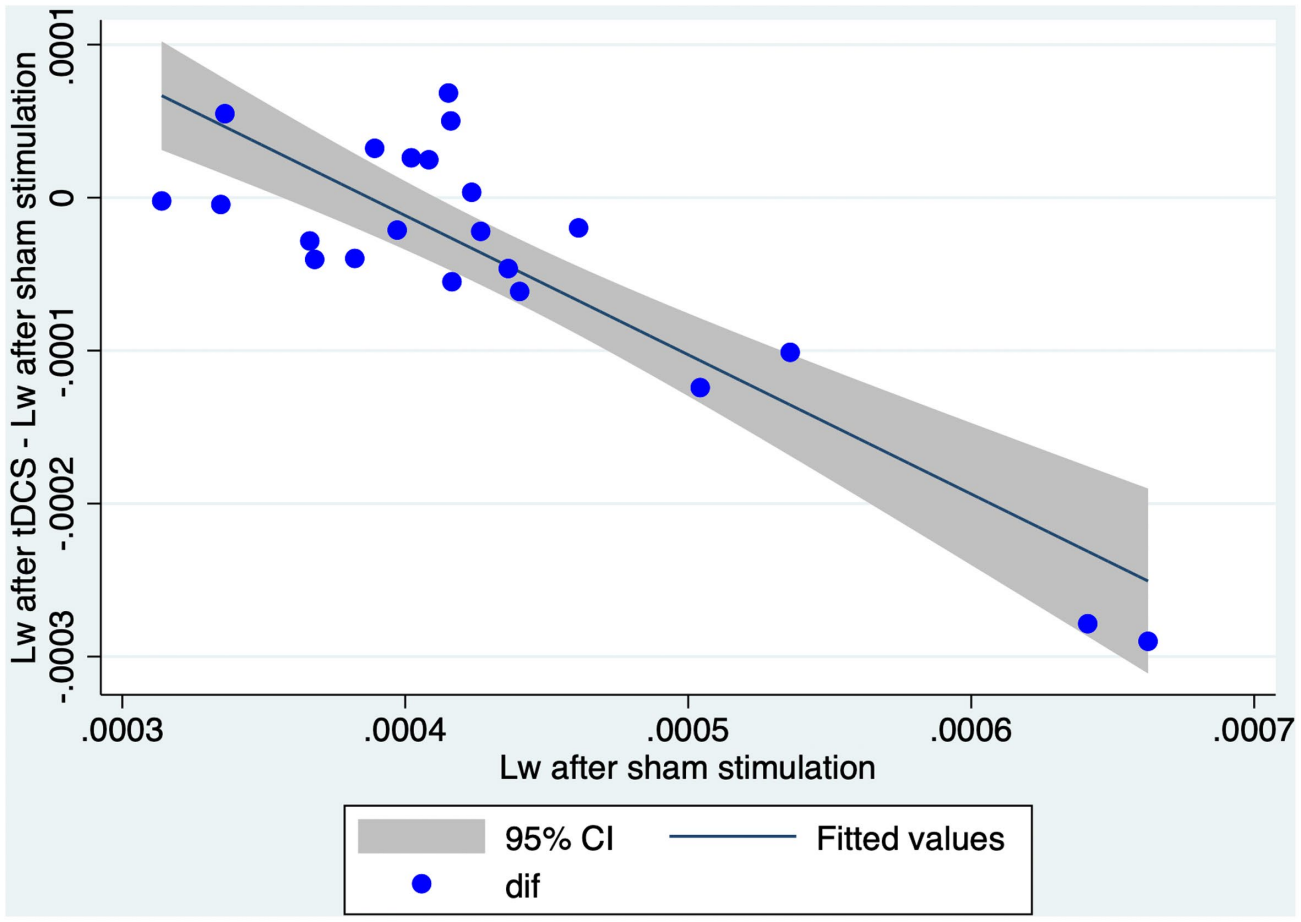
Higher L^w^ after sham condition corresponds to a larger reduction in L^w^ after tDCS. The graph represents a scatter plot of L^w^ after sham stimulation with respect to the differences in L^w^ obtained after tDCS or sham stimulation. Blue points are representative of each individual in the study. CI, confidence interval; L^w^, characteristic path length of the whole brain network; tDCS, transcranial direct current stimulation.

In order to examine the relationship between L^w^ after sham stimulation and the effect of tDCS on L^w^, we performed a simple linear regression. In this model, we calculated the difference in L^w^ between the stimulation conditions (i.e., L^w^ after tDCS - L^w^ after sham) and calculated it based on L^w^ after sham stimulation. A significant regression equation was found [F (1, 20) = 61.34, *p* < 0.01, *R*^2^ = 0.751; Figure 3]. A higher L^w^ after sham stimulation corresponds to a larger reduction in L^w^ after tDCS (Figure 4).

## 4. Discussion

Our main objective was to explore the effect of tDCS (in comparison with sham) on networks of NMDA-receptor-positive GABAergic interneurons. We constructed weighted undirected graphs of the brain networks of 40 Hz ASSR based on dbWPLI and analyzed the effect of tDCS on L^w^ and C^w^. We showed that the L^w^ was significantly lower after tDCS than after sham stimulation. The C^w^ did not differ significantly between the two conditions. These results indicate that the whole brain networks of 40 Hz ASSR become more functionally integrated but not segregated after tDCS simulation. Instead, the effects of tDCS on the properties of the sub-networks (i.e., within right or left frontal, parietal, temporal, and occipital lobes) were not significant. Thus, the effects of tDCS cannot be explained by tDCS-induced changes in the properties of local networks. We also examined whether the effect of tDCS depends on the values of L^w^ after sham condition. A simple linear regression analysis showed that a higher L^w^ after sham condition corresponds to a larger reduction in L^w^ after tDCS, indicating that a larger effect of tDCS is expected for those with a less functionally integrated network of 40 Hz ASSR.

Our study is the first to investigate the effect of tDCS on the L^w^ and C^w^ of a 40 Hz ASSR network. For resting-state networks, however, to the best of our knowledge, only two studies have examined the effects of tDCS on C and L in healthy individuals. Particularly, Mancini et al. (11) recruited 12 adult males and analyzed the effect of a 4-min long tDCS stimulation at 0.6 mA, using an active electrode placed over FC3. They recorded EEG signals during the eyes-open resting condition. They then constructed binary (i.e., applied a range of proportional thresholds) undirected graphs based on dbWPLI and synchronization likelihood and found no significant effect of tDCS on both C and L in any frequency bands. In addition, Vecchio et al. (85) recruited 14 healthy adults and analyzed the effect of 12 min of tDCS at a current intensity of 1.0 mA, using an active electrode placed over C3/C4. They recorded EEG signals during the eyes-closed resting condition, constructed weighted undirected graphs based on lagged linear coherence, and found no significant effect of tDCS on C^w^ or L^w^ in any frequency band. Although it is difficult to compare these results directly because of methodological differences such as the delivery of tDCS (i.e., different current density, duration, and place of electrodes), recording conditions (eyes-open vs. eyes-closed), measures of synchronization (synchronization likelihood, dbWPLI, and lagged linear coherence), and properties of graphs (weighted or unweighted), it is believed that tDCS is not sufficient to induce significant changes in the “resting state” brain network in healthy individuals. Given that tDCS exerts its after-effects by altering the activity of NMDA-receptor positive GABAergic interneurons, and the network of 40 Hz ASSR reflects the function of cortical circuits involving FSPV GABAergic interneurons, the combination of 40 Hz ASSR and graph theory in the present study could be used to detect the after-effect of tDCS, possibly by focusing on this specific neural circuit. Thus, our results provide a framework for describing the neurological underpinnings of the effects of tDCS on cognitive function. Specifically, the changes in cognitive function after tDCS can be explained by its effect on NMDA receptor-positive GABAergic interneurons at the network level. The tDCS functionally integrates this network. Nevertheless, one must be cautious when analyzing the results of L in functional brain networks, as the paths in the graph might not correspond to the information flow (40) and can be tedious to interpret.

Considering the exploratory nature of our analysis examining the “local” effects of tDCS (in relation to sham) on L^w^ and C^w^ in each subgraph of the regions of interest (i.e., right or left frontal, parietal, temporal, and occipital lobes), we opted not to correct for multiple comparisons. This decision preserved statistical power, particularly in the context of small sample sizes or subtle effects, and minimized the risk of Type II errors (i.e., failing to detect a true effect when it actually exists). Despite this less conservative approach, we did not observe a significant effect of stimulation in any brain regions, including the temporal lobes. Traditional views on the 40 Hz ASSR response to an auditory stimulus suggest that the neural activity generating this response is localized to the primary auditory cortex, such as the superior temporal plane (24, 25) or Heschl’s gyrus (26). Subsequently, the “global” effect of tDCS on the whole-brain network of 40 Hz ASSR might stem from its “local” effect on the temporal lobe. However, our study found no significant alterations in the network within the temporal region following tDCS. Recent studies propose that the 40 Hz ASSR is generated by a network of widely distributed brain regions, extending beyond the auditory cortex to frontal and subcortical regions (28), occipital lobe, precentral gyrus, superior parietal lobe (27), parietal, and frontal areas (29). These regions may interact in an organized manner to generate the ASSR (30). Consequently, tDCS could be expected to influence these interactions, leading to changes in the 40 Hz ASSR at the network level. Our study demonstrated a significant “global” effect of tDCS on the whole-brain network. This finding supports the latter viewpoint, suggesting that tDCS can induce subtle, non-significant changes in the frontal lobe and adjacent regions, ultimately altering the interaction of ASSR generators thereby, resulting in a significant change at the network level. However, we must exercise caution in interpreting these results as a non-significant finding does not necessarily imply the absence of an effect. As mentioned in the limitations section, the sample size in this study was small, and a larger sample may be required for higher statistical power. Future research should explore these possibilities and investigate the effects of tDCS on the ASSR network.

Linear regression models were employed to explore the relationship between L^w^ after sham stimulation and the reduction in L^w^ following tDCS. Our results revealed that a larger effect of tDCS (i.e., a more substantial reduction in L^w^ after tDCS) was observed in individuals with higher L^w^ after sham stimulation. This suggests that those with higher L^w^ after sham stimulation are more sensitive to tDCS-induced modulation of brain networks than those with lower L^w^ after sham stimulation. Consequently, our study contributes to understanding the potential effects of tDCS on the 40 Hz ASSR network in healthy individuals. To the best of our knowledge, only Ying et al. (30) examined the graph-theoretical properties of 40 Hz ASSR networks in healthy adults and patients with schizophrenia. They found that patients with schizophrenia had a higher L value than healthy subjects. It is noteworthy that a recent meta-analysis reported that tDCS can ameliorate symptoms of schizophrenia (86). Considering our findings (i.e., reduction of L^w^ after tDCS in a healthy population), one might speculate that tDCS could shorten L^w^ in patients with schizophrenia, bringing their brain networks closer similar to that of healthy individuals. Furthermore, patients with higher L^w^ might potentially benefit more from tDCS intervention, as they might be more sensitive to tDCS-induced modulation of brain networks. However, caution must be exercised when interpreting these results considering their potential implications for clinical applications. Firstly, we did not evaluate the relationship between graph theoretical and behavioral measures. Therefore, a reduction in L^w^ might or might not correlate with therapeutic effects, or, show a correlation with the harmful effects of tDCS. Secondly, it is essential to note that our study involved only healthy participants but not patients with psychiatric disorders; hence, these findings cannot be generalized directly to other populations. Further large-scale studies that include both healthy individuals and those with cognitive or neurological impairments are needed to more conclusively identify the benefits of tDCS and to explore the clinical implications of our findings. This will help evaluate both graph theoretical and behavioral measures in a broader context.

In this study, only male subjects were included to control for potential differences in brain structure and function based on the sex of the participants, which could influence the effects of tDCS on 40 Hz ASSR modulation. Numerous studies have reported that various aspects of brain structure, function, and connectivity differ between sexes. For instance, Ritchie et al. (87) conducted a large sample study of structural and functional differences in the human brain based on sex, discovering that males exhibited higher raw volumes, raw surface areas, and white matter fractional anisotropy, while females displayed higher raw cortical thickness and higher white matter tract complexity. Furthermore, functional brain networks differed between males and females, with stronger connectivity for males in unimodal sensorimotor cortices and stronger connectivity for females in the default mode network (87). In addition to differences in brain structure, function, and connectivity owing to sex, hormonal fluctuations in females due to the menstrual cycle have been hypothesized to produce cyclic alterations in connectivity between the intrinsic networks of the brain (88), which could potentially impact the effects of tDCS on 40 Hz ASSR. Considering the aforementioned differences, we chose to use a homogeneous sample of male subjects to minimize the variability in our results. By doing so, we aimed to provide more reliable and consistent findings on the effects of tDCS on 40 Hz ASSR modulation. Hence, these findings cannot be directly generalized to female subjects, and future studies should investigate the effects of tDCS on 40 Hz ASSR in female populations, taking into account the differences that are sex-dependent and the influence of hormonal fluctuations. This would extend our understanding of tDCS effects on gamma-range ASSRs and their potential therapeutic applications across different populations.

This study had several methodological limitations. Firstly, due to the small sample size (22 participants and 44 observations), the effects might have been overestimated (89). To accurately estimate the effect sizes of tDCS interventions using graph metrics, future studies should employ larger sample sizes. Secondly, we focused only on anodal tDCS at F3. To clarify the region- and dose-specific effects of tDCS, further research examining various types of stimulation (e.g., varied ranges of current intensity, duration of stimulation, and location of the electrodes) is needed. Importantly, all participants were adult males; thus, the present results should not be generalized unless confirmed in females. Additionally, comparing the results of this study with other graph-theoretical studies using different numbers of ROIs (i.e., nodes) may not be appropriate, as graph metrics depend on the number of nodes and edges. This dependence is particularly notable when the number of nodes is less than 200 (90), and no satisfactory methods for correcting this dependency have been reported yet. Another limitation is that we only focused on changes in graph metrics without evaluating their relationship with behavioral measures. Further studies combining graph theoretical and behavioral measurements are needed to examine how changes in the 40 Hz ASSR network affect behavior. Our study required the participants to remain awake and the effect of this task and the click-train stimulus on the network of 40 Hz was likely minimal. Nevertheless, the 40 Hz ASSR network may slightly differ if no additional tasks were performed, warranting further investigation. Moreover, we did not measure the brain’s state before stimulation, which could limit our understanding of state dependency’s impact on the observed tDCS effects. Our study design accounted for brain state fluctuations over months but did not consider shorter fluctuations occurring within hours or seconds. By not measuring the state of the brain before stimulation, we might not have captured the full impact of tDCS effects. This limitation should be considered when interpreting our results and could be addressed in future research by incorporating assessments of brain states before, during, and after stimulation to better understand the role of state dependency in tDCS outcomes. Another concern is the choice of the 30–50 Hz frequency range of interest for capturing the brain network of the 40 Hz ASSR, which is broader than in some previous studies investigating 40 Hz ASSR [e.g., 35–45 Hz (91–94); or 38–42 Hz (95–98)]. Our approach, with this broader frequency range of interest, may increase the sensitivity of detecting the network dynamics of 40 Hz ASSR. However, this strategy could also result in less accurate estimations of 40 Hz ASSR-related network dynamics. Future research should consider employing narrower frequency ranges to gain a more nuanced understanding of the specific contributions of different frequency bands to the observed effects. An important limitation is the lack of control over the time of day during MEG recordings and tDCS sessions. Prior research has shown that circadian rhythms can differentially modulate cortical oscillatory power estimations in sensory regions (99–101). Consequently, the variability in our results could be a side-effect of circadian rhythm fluctuations. Future studies should carefully control the time of day during MEG recordings and tDCS sessions to account for potential circadian influences on cortical oscillations, thereby, enhancing the reliability of the findings and providing a more comprehensive understanding of tDCS effects on brain networks. An additional limitation of the current study is related to our analysis approach. We used linear regression analyzes of task-averaged data to investigate the effects of tDCS on the 40 Hz ASSR network. While this method allowed us to capture the overall effects of tDCS on the network, it did not permit us to evaluate the temporal evolution of network dynamics upon tDCS cessation at a single-trial level. Future studies employing more specific statistical analyzes of connectivity-based parameters across the task block at the single-trial level could provide further insights into the after-effects of tDCS on network dynamics, offering a more comprehensive understanding of the temporal aspects of tDCS-induced changes in brain networks.

In conclusion, our study demonstrated that L^w^ was lower following tDCS compared to sham stimulation, and a higher L^w^ after sham stimulation was associated with a significant reduction in L^w^ after tDCS. These findings suggest that tDCS facilitates functional integration within the 40 Hz ASSR network and that brains exhibiting less integrated 40 Hz ASSR networks after sham stimulation are more susceptible to tDCS modulation. To further elucidate the relationship between the L^w^ of 40 Hz ASSR and behavioral measures, future studies should incorporate both graph theoretical and behavioral assessments. We acknowledge that our study employed a bipolar stimulation protocol, with an anode on one side and a cathode on the other. This configuration might lead to different effects compared to alternative tDCS approaches or electrode placements. Consequently, the observations reported in our study may not necessarily apply to other tDCS configurations, and additional research is required to explore the impact of various tDCS types and electrode locations on 40 Hz ASSR modulation. Given this limitation, our findings should be interpreted cautiously, and future studies should examine the effects of different stimulation parameters and electrode placements to enhance our understanding of the effects of tDCS stimulation on the brain network.

## Data availability statement

The raw data supporting the conclusions of this article will be made available by the authors, without undue reservation.

## Ethics statement

The studies involving human participants were reviewed and approved by the Ethics Committee of Kanazawa University Hospital. The patients/participants provided their written informed consent to participate in this study.

## Author contributions

TT and MKi contributed to the study’s conception and design. YM and YYo organized the database. DS, MKa, and NF performed MEG data processing. TH, MS, KK, and YYa performed the statistical analyzes. TH wrote the first draft of the manuscript. All authors contributed to the manuscript revision and have read and approved the submitted version.

## Funding

This work was supported by the Center of Innovation Program of the Japan Science and Technology Agency. The funder played no role in the study design, data collection, analysis, decision to publish, or manuscript preparation. This research was partially supported by grants from the Moonshot Research and Development Program: grant number JPMJMS2297 from Japan Science and Technology.

## Conflict of interest

The authors declare that the research was conducted in the absence of any commercial or financial relationships that could be construed as a potential conflict of interest.

## Publisher’s note

All claims expressed in this article are solely those of the authors and do not necessarily represent those of their affiliated organizations, or those of the publisher, the editors and the reviewers. Any product that may be evaluated in this article, or claim that may be made by its manufacturer, is not guaranteed or endorsed by the publisher.

## Footnotes

^1^ http://surfer.nmr.mgh.harvard.edu/

^2^ https://neuroimage.usc.edu/brainstorm/tutorials/

^3^ https://sites.google.com/site/bctnet/
