# Supplementary material for: Effect of transcranial direct current stimulation on the functionality of 40 Hz auditory steady state response brain network: graph theory approach
Source: Front Psychiatry. 2023 Jun 9;14:1156617. doi: 10.3389/fpsyt.2023.1156617 (PMC10288104; doi:10.3389/fpsyt.2023.1156617)
Supplement: Supplementary file 3 [file Data_Sheet_1.DOCX]

Supplementary Material

Effect of transcranial direct current stimulation on the functional brain network of 40 Hz auditory steady state response: Graph Theory approach

**Tetsu Hirosawa^1*^, Daiki Soma^2^, Yoshiaki Miyagishi^2^, Naoki Furutani^2^, Yuko Yoshimura^1,3^, Masafumi Kameya^2^, Yohei Yamaguchi^2^, Ken Yaoi^2^, Masuhiko Sano^2^, Koji Kitamura^2^, Tetsuya Takahashi^4^, and Mitsuru Kikuchi^1,2^**

*** Correspondence:** Tetsu Hirosawa: hirosawatetsu1982@yahoo.co.jp

# Supplementary Methods

**The debiased estimation of weighted phase lag index (dbWPLI)**

The oscillatory neuronal activity in different brain regions can be phase-coupled. This is called phase synchronization and has been hypothesized to be a mechanism for creating a flexible communication structure between brain areas (1,2). Traditionally, EEG- or MEG-measured spectral coherence (3) or phase-locking values (4) have been used to quantify phase synchronization. However, indexing MEG-measured phase synchronization can be complicated by (i) the volume conduction of the source activity and (ii) sample-size bias. Volume conduction of source activity can spuriously inflate phase synchronization indices (5) and is especially problematic for EEG or MEG data because of their low spatial resolutions.

To overcome the problem of volume conduction, some indices have been developed. For example, Nolte et al. proposed an imaginary component of coherency (ImC) (5) that is robust to a spurious increase in phase synchronization by the volume conduction of independent sources. However, Stam et al. argued that ImC is not effective at detecting synchronization if the two sources of interest are in or out of phase (6). Stam et al. proposed the phase lag index (PLI), which estimates, for a particular frequency, the extent to which the phase leads and lags between signals from two sensors are non-equiprobable, irrespective of the magnitude of the phase leads and lags. PLI performs better than ImC (6); however, sensitivity of PLI to noise and volume conduction may be hindered by the discontinuity in this index, as small perturbations turn phase lags into leads and vice versa. Importantly, when directly estimated, both the PLI and ImC magnitudes can be positively biased (i.e., sample size bias) (7).

To solve problems of volume conduction and sample-size bias, Vinck et al. introduced an index, namely the weighted phase lag index (WPLI), as well as debiased estimator of the squared WPLI (dbWPLI) (7). The WPLI extends the PLI in that it weighs the contribution of observed phase leads and lags by the magnitude of the imaginary component of the cross-spectrum. In this way, it alleviates the discontinuity of PLI. Furthermore, they proposed to use a debiased estimator of the dbWPLI because WPLI is positively biased when directly estimated. In this way, it alleviates the positive bias of the direct estimator of WPLI (7).

In addition to volume conduction and sample size bias, biases in the synchronization measure should be considered in this study because they may have a direct consequence on the estimation of the network topology (8). Van Diessen et al. discussed in a recent review (9) that estimates of phase synchronization are biased by the epoch length and that the severity of this bias varies for different indices. Haartsen et al. (10) tackled this problem and concluded that using a high number (<90) of short (1000–2000ms) epochs and calculating the dbWPLI via Fourier transform would be most appropriate for characterizing connectivity.

**Calculation of dbWPLI**

The dbWPLI was calculated according to Vinck et al. (7). In particular, we computed (i) the imaginary components of the cross spectra, (ii) the average imaginary component of the cross spectra, and (iii) normalized the computed average over the magnitude of the imaginary component of the cross spectra. In particular, we proceed as following:

Let X≡(X_1_,...,X_N_) denote the observed data vector consists of the measured cross-spectra of two difference signal sources for a particular frequency in N epochs. The _j_-th element of x is defined as

$$X_{j}=Z_{1j}Z_{2j}^{*}$$

where Z_1j_ and Z_2j_ represent the complex Fourier spectra for the frequency observed at each sensor in the jth trial (these values depend on the chosen frequency, but we omit the frequency dependency in our notation because it is always implicitly assumed), and the asterisk indicates a complex conjugate. Then, the dbWPLI is calculated as

$$dbWPLI=\frac{\sum_{j=1}^{N}\sum_{k\neq j}\mathfrak{I\{}X_{j}\mathfrak{\}I\{}X_{k}\}}{\sum_{j=1}^{N}\sum_{k\neq j}\mathfrak{|I\{}X_{j}\mathfrak{\}I\{}X_{k}\}|}$$

where the numerator is the sum of all pairwise products of the imaginary components, and the denominator is the sum of all pairwise products of the magnitudes of the imaginary components.

# Supplementary Tables

**Supplementary Table 1.** L^w^ after tDCS and sham stimulation of the whole brain network and subgraphs of each brain region.

Values for L^w^ after tDCS and sham stimulation are expressed as mean ± s.d.; The asterisks indicate statistically significant differences as measured by the linear mixed models (p < 0.05.)

**Supplementary Table 2.** C^w^ after tDCS and sham stimulation for the whole brain network and subgraphs of each brain region.

Values for C^w^ after tDCS and sham stimulation are expressed as mean ± s.d.;

The asterisks indicate statistically significant differences as measured by the linear mixed models (p < 0.05.)

# Supplementary References

1. Engel AK, Fries P, Singer W. Dynamic predictions: oscillations and synchrony in top–down processing. Nat Rev Neurosci (2001) 2:704–16. doi: [**10.1038/35094565**](https://doi.org/10.1038/35094565).

2. Varela F, Lachaux JP, Rodriguez E, Martinerie J. The BrainWeb: phase synchronization and large-scale integration. Nat Rev Neurosci (2001) 2:229–39. doi: [**10.1038/35067550**](https://doi.org/10.1038/35067550).

3. Walter DO. Spectral analysis for electroencephalograms: mathematical determination of neurophysiological relationships from records of limited duration. Exp Neurol (1963) 8:155–81. doi: [**10.1016/0014-4886(63)90042-6**](https://doi.org/10.1016/0014-4886(63)90042-6).

4. Lachaux JP, Rodriguez E, Martinerie J, Varela FJ. Measuring phase synchrony in brain signals. Hum Brain Mapp (1999) 8:194–208. doi: [**10.1002/(sici)1097-0193(1999)8:4<194::aid-hbm4>3.0.co;2-c**](https://doi.org/10.1002/(sici)1097-0193(1999)8:4%3c194::aid-hbm4%3e3.0.co;2-c).

5. Nolte G, Bai O, Wheaton L, Mari Z, Vorbach S, Hallett M. Identifying true brain interaction from EEG data using the imaginary part of coherency. Clin Neurophysiol (2004) 115:2292–307. doi: [**10.1016/j.clinph.2004.04.029**](https://doi.org/10.1016/j.clinph.2004.04.029).

6. Stam CJ, Nolte G, Daffertshofer A. Phase lag index: assessment of functional connectivity from multi channel EEG and MEG with diminished bias from common sources. Hum Brain Mapp (2007) 28:1178–93. doi: [**10.1002/hbm.20346**](https://doi.org/10.1002/hbm.20346).

7. Vinck M, Oostenveld R, van Wingerden M, Battaglia F, Pennartz CM. An improved index of phase-synchronization for electrophysiological data in the presence of volume-conduction, noise and sample-size bias. NeuroImage (2011) 55:1548–65. doi: [**10.1016/j.neuroimage.2011.01.055**](https://doi.org/10.1016/j.neuroimage.2011.01.055).

8. van Wijk BCM, Stam CJ, Daffertshofer A. Comparing brain networks of different size and connectivity density using graph theory. PLOS ONE (2010) 5:e13701. doi: [**10.1371/journal.pone.0013701**](https://doi.org/10.1371/journal.pone.0013701).

9. van Diessen E, Numan T, van Dellen E, van der Kooi AW, Boersma M, Hofman D, et al. Opportunities and methodological challenges in EEG and MEG resting state functional brain network research. Clin Neurophysiol (2015) 126:1468–81. doi: [**10.1016/j.clinph.2014.11.018**](https://doi.org/10.1016/j.clinph.2014.11.018).

10. Haartsen R, van der Velde B, Jones EJH, Johnson MH, Kemner C. Using multiple short epochs optimises the stability of infant EEG connectivity parameters. Sci Rep (2020) 10:12703. doi: [10.1038/s41598-020-68981-5](https://doi.org/10.1038/s41598-020-68981-5).
